# StructuRly: A novel shiny app to produce comprehensive, detailed and interactive plots for population genetic analysis

**DOI:** 10.1371/journal.pone.0229330

**Published:** 2020-02-19

**Authors:** Nicola G. Criscuolo, Claudia Angelini

**Affiliations:** 1 Department of Environmental Systems Science, ETH Zürich, Zurich, Switzerland; 2 Istituto per le Applicazioni del Calcolo “M. Picone”, National Research Council, Naples, Italy; Imperial College London, UNITED KINGDOM

## Abstract

Population genetics focuses on the analysis of genetic differences within and between-group of individuals and the inference of the populations’ structure. These analyses are usually carried out using Bayesian clustering or maximum likelihood estimation algorithms that assign individuals to a given population depending on specific genetic patterns. Although several tools were developed to perform population genetics analysis, their standard graphical outputs may not be sufficiently informative for users lacking interactivity and complete information. StructuRly aims to resolve this problem by offering a complete environment for population analysis. In particular, StructuRly combines the statistical power of the R language with the friendly interfaces implemented using the shiny libraries to provide a novel tool for performing population clustering, evaluating several genetic indexes, and comparing results. Moreover, graphical representations are interactive and can be easily personalized. StructuRly is available either as R package on GitHub, with detailed information for its installation and use and as shinyapps.io servers for those users who are not familiar with R and the RStudio IDE. The application has been tested on Linux, macOS and Windows operative systems and can be launched as a shiny app in every web browser.

## Introduction

Population genetics studies focus on the genetic variation within and among populations by examining the distributions and the changes over time in the frequencies of genes and alleles, which may occur in different forms (polymorphism) [1], and by the estimation of the overall genetic diversity of individuals present inside populations. In recent years, the analysis of genetic markers such as restriction fragment length polymorphisms (RFLPs), amplified fragment length polymorphisms (AFLPs), microsatellites (SSR), and single-nucleotide polymorphisms (SNPs) has been usually carried out through algorithms based on Bayesian clustering and maximum likelihood implemented, respectively, in software as *STRUCTURE* [2] and *ADMIXTURE* [3], which represent some of the most remarkable examples, among many others. The primary aim of this type of population genetic software is to describe the population structure that best represents the genetic characteristics of the individuals under study by estimating the best number of clusters (*K*) (i. e., sub-populations) in which to divide the collected samples [4], identifying group-specific genetic pattern/distribution and assigning each individual to the sub-population with better fit. However, given the information that a user can specify at the beginning of population analysis (i. e., sample identifiers (IDs), putative populations defined a priori by the researcher and sampling sites), the standard graphical outputs of these tools, such as the well-known *STRUCTURE* barplot, may not always be thoroughly informative since they do not allow to use and visualize the available additional information.

In recent years, the development of open-source software specially designed for the production of high-quality and interactive plots is becoming very active in various scientific fields [5–7]. Novel open source applications have also been developed to customize the graphical output of the Bayesian analysis [8]. Nevertheless, no attempt has yet been made to display information that population software might exploit to improve the interpretation of cluster analysis, such as the collection sites [9], or general information such as the IDs of the samples or the putative populations defined by the user. Moreover, a population cluster analysis is often combined with the estimation of specific genetic diversity indices (e. g. Shannon-Wiener, Simpson or Stoddart), with the visualization of the samples through the Principal Coordinates Analysis (i. e., the classical multidimensional scaling) and with the comparison of the results obtained with other clustering methods implemented in other software [10–13]. Retrieving, organizing, and inspecting such information usually requires combining several tools. A typical post-analysis scenario puts the researcher engaged in the simultaneous use of different software to produce outputs not always immediately comparable, with the consequence that it is necessary to carry out a supplementary and time-consuming exporting job of the graphic and numerical components of a precise output. Just after that, generally, a third software for comparing results is exploited (as R, given the great availability of open source packages to produce detailed graphs and to calculate different genetic indexes). With StructuRly, the user will have a single application to carry on complete population analysis.

## Materials and methods

StructuRly is a novel R package [14] implementing a user-friendly graphical interface that offers an inclusive environment for the analysis of genetic data, the customization, and the organization of results. The user interface has been implemented using the *shiny* package [15], the computational core consists of the functionalities of *ade4*, *adegenet*, *mcclust*, *pegas*, and *poppr* R packages, and the graphical part of the application has been implemented through the functionalities of the *ggplot2* package [16]. Moreover, StructuRly exploits the functions of the *plotly* package [17] to generate interactive plots with intuitive added features, giving the users the possibility to zoom, isolate only certain classes of samples and obtain essential information by pointing the cursor on a specific part of the graph. Besides, all the figures can be downloaded as high-resolution images in different formats. Finally, StructuRly also uses the new *shinymeta* package that allows the user to download the source code that has been used to generate a given figure.

## StructuRly main interface

**Fig 1:**
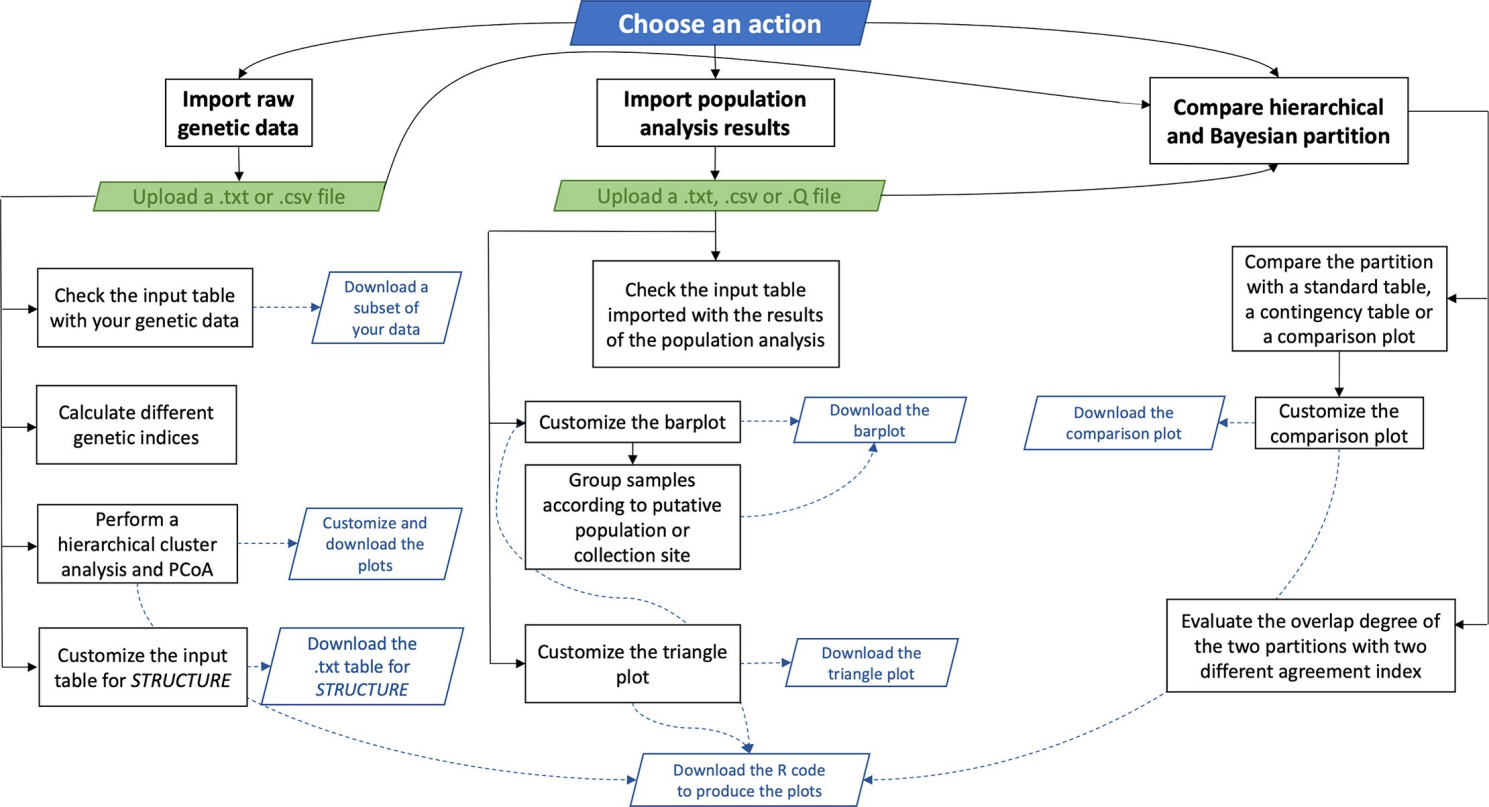
StructuRly internal modular organization. StructuRly consists in three main sections. The first two sections of the application (‘Import raw genetic data’ and ‘Import population analysis results’) are independent and are based on the two different types of input files that can be imported by the user, i. e., genetic raw data or the output of other population analysis software such as *STRUCTURE* or *ADMIXTURE*, respectively. The third section (‘Compare hierarchical and Bayesian/maximum likelihood estimation partition’) can be activated after having processed the data within the first two modules to compare partitions obtained from two different types of cluster analysis.

**Fig 2:**
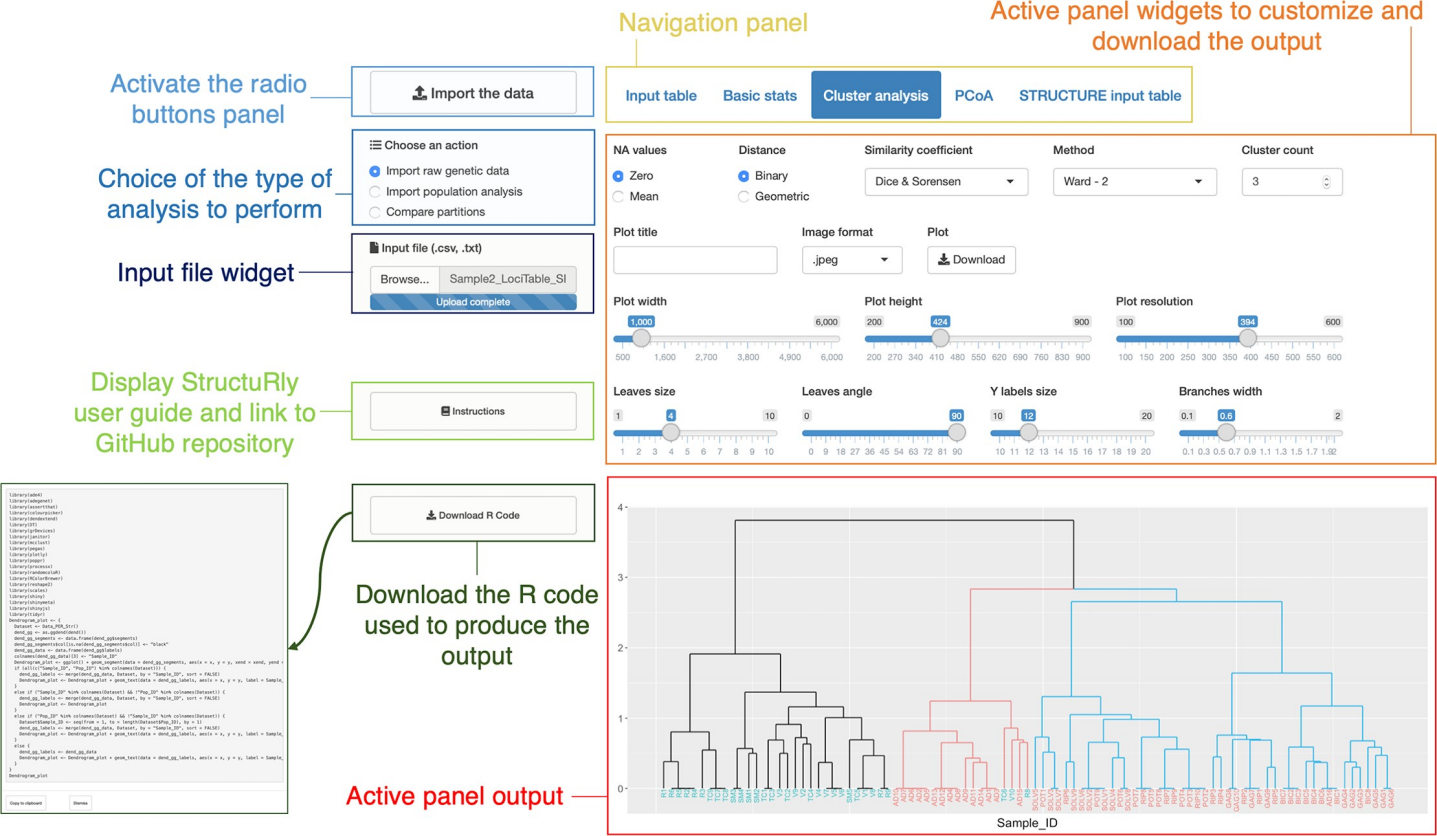
Main components of the StructuRly UI. The left side command column is static and allow the user to control which section to activate by loading the appropriate input file into the software. Three sections are now available. Once one of the sections is activated, a more specific interface is visualized in the right side. The buttons on the upper navigation panel are specific of each section and are used to activate different sub-sections according to the type of output to produce. Moreover, in the sections where it is possible to produce a graphical output, a special button allow to download the R code used to produce the plot.

We implemented StructuRly as a modular software schematically represented in Fig 1. Each time StructuRly is executed by typing *runStructuRly()* in the R console or by visiting its website on shinyapps.io servers, the main interface is opened. First of all, the user has to import the data. To this purpose, he/she needs to press the import data button. Then, from the “choose an action” panel (on the left side of the primary user interface, as shown in Fig 2), he/she can activate one of the three main sections illustrated in Fig 1 depending on the type of the input file to be analyzed (in .txt, .csv or .Q and .fam format) or the comparison one is interested in. Genetic raw data and output from other tools must be suitably formatted before being processed (on the GitHub page, there are detailed information about data format). After that, the user has to press the start analysis button. The data are uploaded, and the specific user interface becomes active. From the input table panel, the user can verify that the data are correctly loaded, or if it is required to modify the column separator, or the quotes.

The user interface (UI) structure is similar in all sections, and it is divided into many sub-panels based on the type of output (tabular or graphical) to produce (see Fig 2 and additional description in Supplementary material).

In the first section of StructuRly, the user can import a file containing raw genetic data. Here, it is possible to carry on a first exploration of the population structure by calculating different genetic indices (sub-panel Basic Stats), performing hierarchical cluster analysis based on geometric distance measures or similarity coefficients and several linkage methods (sub-panel Cluster analysis), and visualizing the sample in the principal coordinates space (sub-panel PCoA). It is also possible to visualize that table (sub-panel Input Table) and format it as input for *STRUCTURE*.

In the second section, the user can import the data tables with the results obtained from popular cluster analysis software, such as *STRUCTURE* and *ADMIXTURE*, to depict them on a fully customizable, interactive, and colorful barplot and triangle plot. Moreover, the barplot implemented in StructuRly allows the user to group the samples according to the putative populations, the collection sites, or according to both.

Finally, in the third section, the user can compare the results obtained in the first and the second section. In particular, it is possible to compare the partition of the hierarchical and Bayesian cluster analysis through a standard contingency table and an interactive heatmap. In this section, the Rand Index (RI) and the Adjusted Rand Index (ARI) [18, 19] give a measure of the degree of similarity between the two partitions [20].

## Software availability and requirements

StructuRly is freely available on GitHub at https://github.com/nicocriscuolo/StructuRly as an R package; the installation requires R (> = 3.5) and RStudio (> = 1.2). Moreover, it is available on the shinyapps.io servers for those users who are not familiar with the RStudio IDE. StructuRly is available online at the following link: https://nicocriscuolo.shinyapps.io/StructuRly/. We tested both local and online versions on macOS, Windows, and Linux operative systems.

S1 Fig shows a graphical step-by-step tutorial on how to obtain the desired output.

More information about the functionalities available in each section, data format specification, requirements, main interface, and the user manual are available in the same GitHub repository, where it is also possible to download sample datasets to understand StructuRly usage and reproduce all the plots shown in this work. Moreover, at https://youtu.be/0FUFM6GNaYI, there is a video tutorial describing StructuRly functionalities.

## Results and discussion

StructuRly provides the user with a complete environment for population analysis based on molecular markers characterized by weight in terms of base-pairs (bp), as microsatellite, SSR, AFLP, RFLP. The three different sections allow the user to perform the analysis of raw genetic data, produce and organize specific and detailed interactive plots from the output of other population software, such as *STRUCTURE* and *ADMIXTURE*, and compare partitions obtained using different clustering methods. Moreover, for every section, StructuRly exploits the functions of the *shinymeta* package to allow the download of the R code implemented within the Shiny application and that has been used to produce the graphical outputs. This feature offers to the users the possibility to easily share and reproduce their research.

In the following, for each section, we illustrate the advantages of StructuRly by considering a whole population analysis process based on multi-locus genotype data.

Sample datasets are available on the GitHub repository and consist of .txt, .csv or .Q and .fam files. Each dataset can contain one or more variables with sample information (such as the sample ID or the name of the sampling site, or both). For each locus, the dataset with the raw genetic data contains as many columns as the organism’s ploidy. Similarly, the one with the population analysis results consists of as many columns as the number of clusters chosen for the analysis.

The results illustrated in the following sections are representative of a typical usage scenario of StructuRly. By analyzing the data genetic data of the “Sample2” group downloadable on the GitHub repository, it is possible to reproduce all the figures present in this manuscript. These raw genetic data represent the weight in bp of microsatellite genetic loci (SSR) collected in a subset (95 individuals) of *Olea europaea* L. specimens (a diploid organism) described in Criscuolo et al. [10].

## Section–Import raw genetic data

At the beginning of the analysis, the user can import the raw genetic data in .txt and .csv format. After specifying the characteristics of his table as the proper column separator (e. g. ‘comma’, ‘tab-delimited file’, and/or the presence/absence of quotes), it is possible to compute several genetic indices and perform a hierarchical cluster analysis using both genetic distance or binary similarity with different types of linkage methods. In particular, once the raw data are imported, StructuRly displays them as an interactive table-form. It is possible to re-format such a table to produce a structured .txt file to be directly imported into the *STRUCTURE* software to perform the Bayesian analysis [9].

**Fig 3:**
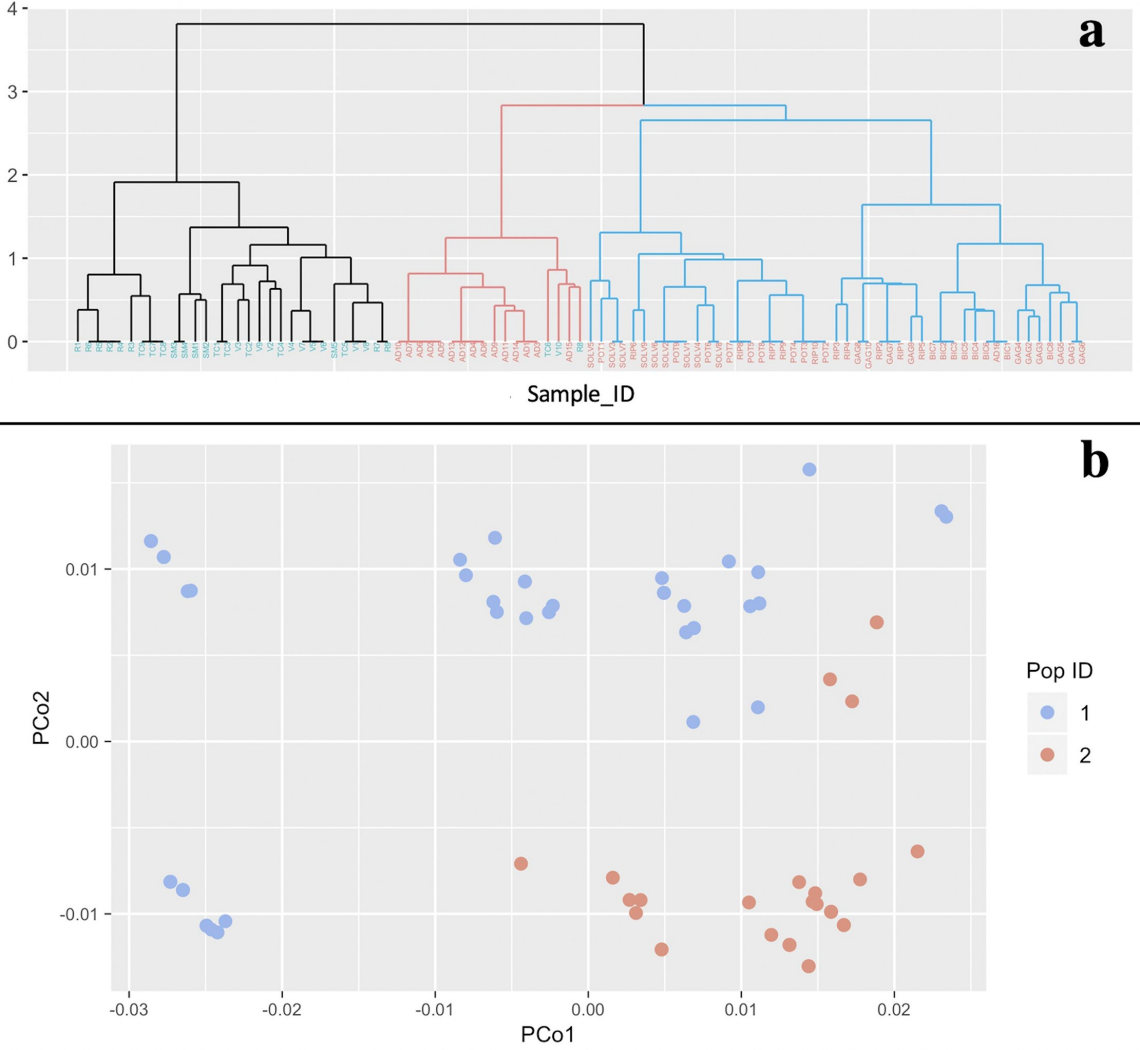
StructuRly graphical outputs of the hierarchical cluster analysis and the PCoA. From the “Import raw genetic data” section, the user can produce (a) the dendrogram based on a hierarchical cluster analysis, and (b) a Principal coordinates analysis based on different dissimilarity indices.

As an example, by considering the *Olea europaea* data, and loading the file Sample2_LociTable_SP.csv into StructuRly, the user can immediately compute a hierarchical cluster analysis. StructuRly offers the possibility to choose both geometric distances (e. g. Euclidean, Manhattan, ecc,) or binary similarity matrices (e. g. Jaccard, Dice-Sørensen, etc.) to calculate a matrix of distances to be subjected to cluster analysis with eight different types of linkage methods, including Ward and the UPGMA method, widely used in population analysis because they are the ones that best capture genetic groupings between individuals [21]. Fig3A shows the output of this analysis obtained when choosing the Dice-Sørensen distance method and the Ward linkage method. Depending on additional information, the dendrogram can show the result of the cluster analysis through the different colors of the branches, and the putative population through the colors of the leaves reporting the IDs (see Fig 3A).

Similarly, inside the “PCoA” panel, the user can compute a multidimensional scaling of his raw data based on several dissimilarity indices used to calculate a genetic distance, and then produce the graphical output presented in Fig 3B. Similarly, it is possible to perform the PCoA also on the results of population cluster analysis, i. e. by importing in the first section of StructuRly a dataset created for the second section. Finally, the application allows the download of all the plots mentioned above in different high-quality formats.

Moreover, StructuRly can perform the first level of population analysis by calculating a series of statistics on the genetic loci under investigation, such as the type and allele frequency and missing values (S3 Fig). After that, StructuRly can compute several genetic diversity indexes that give a measure of how many different loci are present in a given population, such as Shannon-Wiener, Simpson, and Stoddard [22–24], expected heterozygosis, richness, and *p*-gen, the probability of finding a genotype identical to that present in an individual under the hypothesis of sexual reproduction and random breeding [25]. A more detailed level of analysis on the set of available loci can be carried out by also calculating the Hardy-Weinberg equilibrium, obtaining both the standard and the exact *p*-value (the latter one for diploid organisms) based on an arbitrary number of permutations [26].

Finally, StructuRly automatically re-format the genetic data table according to the requirements of the *STRUCTURE* software.

## Section–Import population analysis

In this section, the user can customize the plots typically produced using *STRUCTURE* and *ADMIXTURE* by importing their output files directly in StructuRly. These tools compute the probability that the genotype of an individual belongs or not to a specific sub-population whose number is defined a priori by the user and allow the user to produce the well-known barplot and triangle plot.

StructuRly displays the genetic admixture degree through an interactive barplot. For the first time, if the user has specified necessary sample information in the input file, there is the possibility to visualize the ID of each sample and decide whether to group the individuals according to the initial putative population or the collection site. After customizing the plot (size, resolution, fonts size), the user can choose to color the groups with a customized or a colorblind-friendly palette (a grayscale) and then download the final plot in different formats. On a single image, it is now possible to display up to three additional types of sample information unlike the standard barplot of allele frequencies, in particular, one can visualize:

- IDs;
- putative population which results as a different color of the barplot labels;
- collection site as different symbols present on each bar.

**Fig 4:**
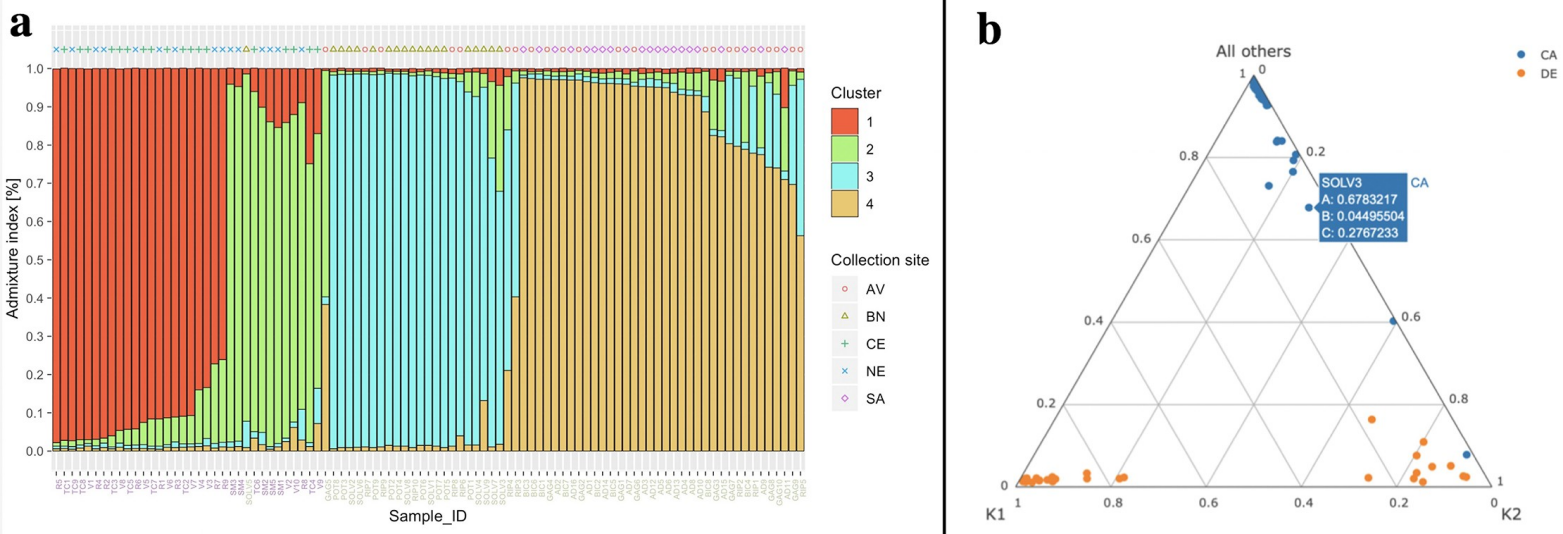
StructuRly graphical outputs of the population cluster analysis. The interactive barplot (a) and the triangle plot (b) are analogous to those obtained as *STRUCTURE* software outputs. They show the results of the population analysis. In StructuRly these plots are interactive, and therefore it is possible to obtain more information on the samples quickly.

For example, considering the olive data, if the user loads in the second section of StructuRly the Sample2_StructureClusters_SPL.csv dataset, he/she can obtain the barplot shown in Fig 4A and the triangle plot shown in Fig 4B. As all other StructuRly plots, the widgets allow to customize several characteristics of the plots as shown in S2 Fig.

To better underline the differences, S4 Fig shows the comparison between the standard *STRUCTURE* barplot (S4A Fig) and the one produced by StructuRly (S4B and S4C Fig) using the same genetic dataset. Thanks to the functionalities of the *ggplot2* package, StructuRly can produce a barplot that contains detailed information regarding the names of the samples, the putative populations assigned by the user and the sampling sites. As an alternative to showing information on a single graph, in StructuRly the user can also divide the samples according to the initial populations or sampling sites, or for both (S4C Fig). In this way, the user can compare the results obtained through information that may support genetic population analysis.

The input files to load in this section are in .txt, .csv and .Q format. Note that the file in .Q format is the standard output obtained using *ADMIXTURE* software. Once uploaded, this file is automatically restructured in tabular format, and the user can subsequently choose to add a file containing basic information about his samples, all in a single work environment and without having to know a programming language.

## Section–Compare partitions

**Fig 5:**
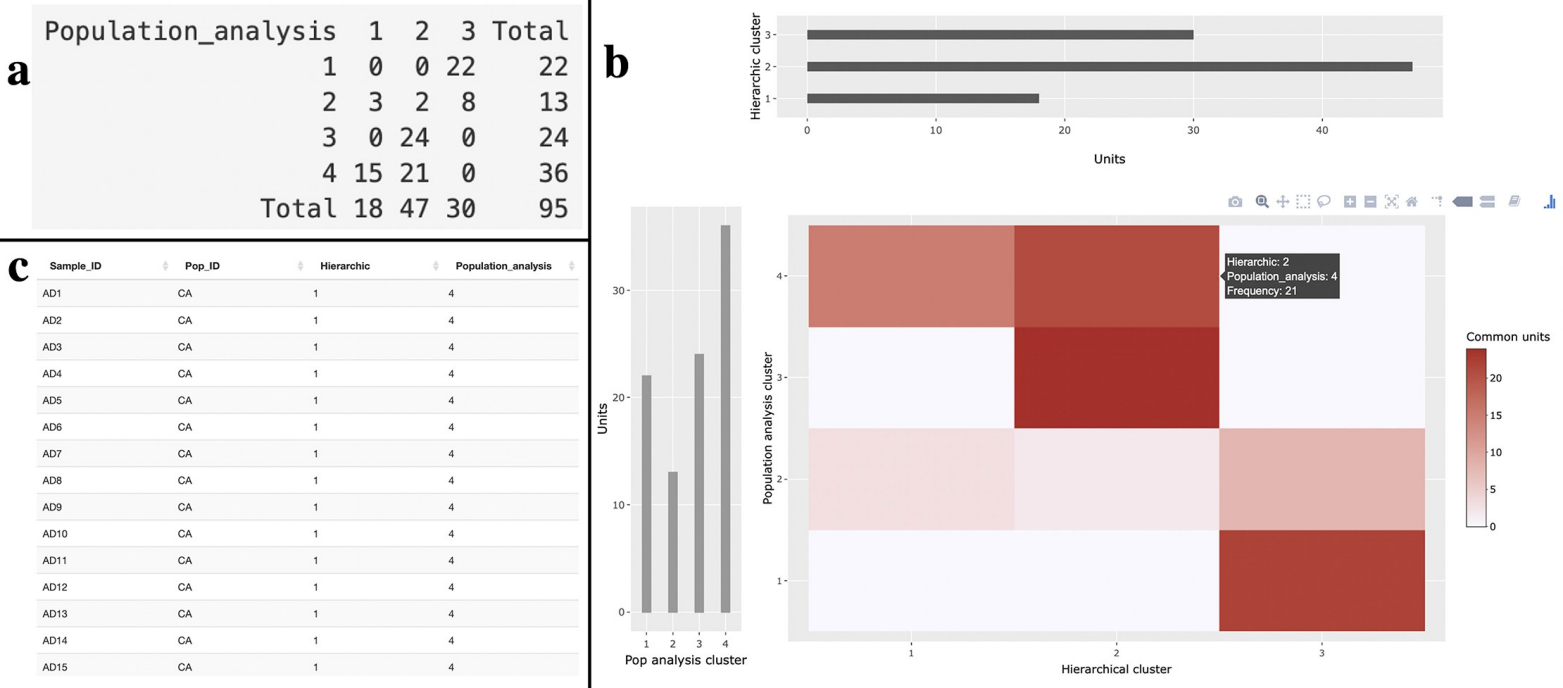
Comparison section outputs. Contingency table (a), standard table (b) and contingency heatmap with two barplots showing the number of units present in inside the partitions obtained respectively from the hierarchical cluster and Bayesian/maximum likelihood estimation analysis (c).

Finally, the third section allows comparing the partition of the hierarchical cluster analysis with the one of the Bayesian (*STRUCTURE*) and maximum likelihood analysis (*ADMIXTURE*) imported in the second sections by visualizing the units belonging to each cross-partition as a contingency table and computing some summary statistics (Fig 5). For the hierarchical cluster analysis, the user can select the number of clusters in which to divide his population directly within section “Import raw genetic data”. For the external population analysis software, the user can choose the best number of groups obtained from the Bayesian analysis during the analysis with *STRUCTURE* and from the maximum likelihood estimation analysis with *ADMIXTURE*. In section “Import population analysis”, StructuRly assigns each individual to the cluster with the maximum value of the posterior probability. The partition obtained in this way is then compared with the hierarchical partition. The user can evaluate the degree of overlap between the two partitions with a standard table, a contingency table, and an interactive heatmap, whose color intensity is directly related to the number of common units within two groups in the different partitions. The degree of overlap can be expressed through the RI or the ARI.

For example, the outputs of the third section of StructuRly produced with the SSR olive data clearly show that the hierarchical cluster analysis and the *STRUCTURE* analysis produced almost the same results because most of the units present in a specific cluster of the hierarchical partition are also present in a defined cluster of the partition obtained through a Bayesian approach (Fig 5A and Fig 5B). However, the labels of the clusters might not necessarily be the same.

## StructuRly and other available tools

StructuRly is an interactive software that allows users to perform a population analysis based on molecular markers characterized by weight in terms of bp.

The first significant advantage in terms of implementation lies in the fact that StructuRly is based on the R language, an open-source programming language, for which thousands of statistical packages are continuously released, updated and tested by a global community. Moreover, StructuRly has a modular architecture (i. e., section/and sub-panels). Thus, it is straightforward to integrate novel functionalities (i. e., adding a new panel or sub-panel), and such choice also facilitates its maintainability. Other programs, such as *STRUCTURE* itself, were developed through other programming languages (e. g. Java), for which such a broad set of open-source statistical analysis packages is not available and are also closer in terms of user customization.

In terms of overall output, most of the results that can be obtained using StructuRly, could also be obtained using other tools (with more effort, lower graphical quality and customization). For example, as shown in section Results, StructuRly can be extremely useful when there is the need to perform a complete population analysis using specific molecular markers to evaluate, for example, the degree of genetic polymorphism within a population [10, 11, 27, 28]. Indeed, this type of analysis usually requires the simultaneous use of different software, such as *GenAlEx* [29], which exploits spreadsheet macros. Therefore, a complete analysis translates into the use and exchange of information between different software, which could both slow down the analysis, but also generate reproducibility problems of the results obtained. Instead, StructuRly provides a single work environment that calculates genetic indices, performs a hierarchical cluster analysis, a principal coordinate analysis, and compares different grouping methods, also generating files ready for a new analysis in a population software such as *STRUCTURE*. For such reasons, our application is also relatively different from open source software which have the only purpose to customize the classic *STRUCTURE* barplot [8]. Our software also offers the user a series of new interactive functions to query the outputs more quickly, view the names of the individual samples and their initial groupings based on the putative population and the sampling site, as well as generate colorblind-friendly plots, significantly differentiating it from software like *pong* [30] and *CLUMPAK* [31] that produce static output. Moreover, StructuRly has a friendly graphical interface that allows also non-expert users to perform population analysis studies, whereas *pong* and *CLUMPAK* works from command line, hence requiring the user to understand the correct use of a programming language.

Finally, StructuRly allows the user to download the source code used to create each graphic output, thus giving the user the possibility to reproduce the results obtained in the software. To the best of our knowledge, no other software has such a feature.

On the other hand, StructuRly also has some limits. This first version allows the users to quickly work only with certain types of molecular markers, thus differentiating from other software, such as PLINK (https://www.cog-genomics.org/plink/1.9/) which handle Next Generation Sequencing (NGS) data as input. Despite that, the analysis of NGS data is supported by several R/Bioconductor packages. Therefore, the modular design user in the development of our Shiny application could, in the future, allow the implementation of novel functionalities that handle these types of experimental data.

## Conclusions

StructuRly was designed to support researchers in population analysis. The interactive application incorporates several tools in a single environment to obtain essential information on population structure without requiring the knowledge of a programming language. For the first time, this application allows the users to obtain publication-ready plots that contain more detailed information on the result of population analysis.

Moreover, since StructuRly works with straightforward pre-formatted data files, its use can be easily extended to other population analysis software such as TESS or TESS3 [32] that are becoming popular in the scientific community. Additionally, StructuRly allows extending its functionalities to different genetic markers, expressed as integers representing the weight of genetic loci in bp. Finally, the modular structure of the application offers, in the future, the possibility to implement new types of graphical outputs, for example by importing new samples information (e. g. geographic coordinates) and the boundaries of sampling site (in shapefile format, .shp) to display the results of the cluster analysis of the genetic markers on a geographical basis (10, 12).

## Supporting information

S1 File StructuRly_SM. This file contains additional information regarding the StructuRly user interface and the supplementary figures.
(DOCX)

S1 Fig (TIFF)

S2 Fig (TIFF)

S3 Fig (TIFF)

S4 Fig (TIFF)
