# Correction: StructuRly: A novel shiny app to produce comprehensive, detailed and interactive plots for population genetic analysis

**DOI:** 10.1371/journal.pone.0329195

**Published:** 2025-07-23

**Authors:** Nicola G. Criscuolo, Claudia Angelini

In the Abstract section, there is a link error in the seventh sentence of the paragraph. The correct sentence is: StructuRly is available either as R package on GitHub, with detailed information for its installation and use and as shinyapps.io servers for those users who are not familiar with R and the RStudio IDE.

In the Software availability and requirements section, there is a link error in the third sentence of the first paragraph. The correct sentence is: StructuRly is available online at the following link: https://nicocriscuolo1618.shinyapps.io/StructuRly/.
