# Supplementary material for: StructuRly: A novel shiny app to produce comprehensive, detailed and interactive plots for population genetic analysis
Source: PLoS One. 2020 Feb 19;15(2):e0229330. doi: 10.1371/journal.pone.0229330 (PMC7029954; doi:10.1371/journal.pone.0229330)
Supplement: S1 File — This file contains additional information regarding the StructuRly user interface and the supplementary figures. (DOCX) [file pone.0229330.s001.docx]

Supplementary material for “StructuRly: a novel shiny app to produce comprehensive, detailed and interactive plots for population genetic analysis”

Nicola G. Criscuolo^1^, Claudia Angelini^2^

^1^Department of Environmental Systems Science, ETH Zürich, Zurich, Switzerland

^2^ Istituto per le Applicazioni del Calcolo “M. Picone”, National Research Council, Naples, Italy

**StructuRly User Interface (UI)**

The StructuRly UI illustrated in Fig 2 of the manuscript is intuitive and straightforward. It is organized into two main parts.

The left column elements are static, and therefore always in the foreground throughout the use of the application. From this column, the radio buttons of the “Choose an action” widget allow deciding which of the three sections to activate and, as a consequence, which widget to show for data import. Selecting “Import raw genetic data” will open the first section, “Import population analysis” the second, and “compare partitions” the third. The lower “Instructions” button visualizes the graphical step-by-step tutorial of the application (S1 Fig) aimed to guide the user in their analysis. The detailed user manual is available on the GihHub page. Finally, thanks to the novel *shinymeta* package, for every graphical output obtained with StructuRly, the bottom “Download R code” button allows the users to download the R code used to produce such output.

The right part of the UI depends on the specific section/sub-section that is activated (once the input file has been imported). However, the organization of such part of the UI is similar for each StructuRly sub-section. On the top, there is always a navigation panel that allows activating different panels. Each panel allows to perform a specific analysis or produce charts of different nature. Once a specific panel is activated, a series of widgets are shown in the upper part of the section, whereas the outputs are shown in the bottom part (usually in graphical form). The widgets allow modifying different characteristics of the graph or the analysis.

After performing the analysis, it is possible to download the plots in different formats, including vectorial types. As shown in S2 Fig, for each sub-panel, there are many widgets to completely customize graphs such as the barplot, which is the primary output of a population cluster analysis (which could be either obtained from *STRUCTURE* or *ADMIXTURE*). These widgets control both the interactive graph, shown directly online on the default web browser, and some features of the outputs available for download (such as graphical resolution).

**Data formats and data scaling-up**

StructuRly allows handling different types of data formats (as detailed described in the "Data input" section of the online user manual). In particular, the user can choose the file type, the column separator, and also perform basic format conversion operations.

The amount of data that StructuRly can handle depends on the specific software section and the user hardware characteristics. We tested the capabilities of the application on a MacBook Pro (2016) with 8 Gb of RAM and a 2.9 GHz i5 Intel Core processor.

Generally speaking, when reading the data and performing fundamental analysis steps, the user should not observe any delay in the reactivity of the app till the limit of about 100,000 elements (as rows × columns). After such a limit, the user may experience some reactivity delays, but it will still be possible to fully exploit some non-graphic functions, like the ones that calculate the diversity indices and the one to re-structure and download the loci datasets to import it in *STRUCTURE*.

On the other hand, when concerning the data visualization, datasets with more than 5,000 units may cause some issues in the visualization of some plots such as the dendrogram, the barplot, or the comparison plot. To avoid label overlap, the users should significantly increase the plot's width and simultaneously reduce the X-axis label size, but this could make it hard to read the analysis results on a graph accurately. A more performing system and a powerful graphics unit can move towards such limits.

However, several improvements can be incorporated in future versions of StructuRly to allows handling larger dataset sizes and more data formats, either by using more efficient functions and fully exploiting parallel implementation.

**S1 Fig.** **Graphical step-by-step tutorial.** The scheme illustrates the different results that is possible to produce in each section of StructuRly following a step-by-step approach aimed to guide the user towards the use of the software. The blue arrows illustrate how to move within the first section, red arrows are devoted to the third section, and green arrow to the third. The left side black points illustrates how to switch between panels

**S2 Fig.** **StructuRly UI of the barplot sub-section.** Example of StructuRly barplot and widgets available to customize the plot.

**S3 Fig**. Example of StructuRly table with several genetic indexes

**S4 Fig.** **Comparison between two software outputs.** Visual comparison between the barplot obtained from the *STRUCTURE* software (a) and the one obtained in StructuRly (b, c). Even though sample information and the putative population are specified in the input file to import into *STRUCTURE*, the output does not show this information directly on the barplot. Instead, the barplot produced with StructuRly is entirely interactive and once downloaded it can show up to three different types of information on a single graph: the name of each sample colored for the initial putative population and the sampling site shown above the plot bars.
