# Supplementary figures and images for: StructuRly: A novel shiny app to produce comprehensive, detailed and interactive plots for population genetic analysis
Source: PLoS One. 2020 Feb 19;15(2):e0229330. doi: 10.1371/journal.pone.0229330 (PMC7029954; doi:10.1371/journal.pone.0229330)

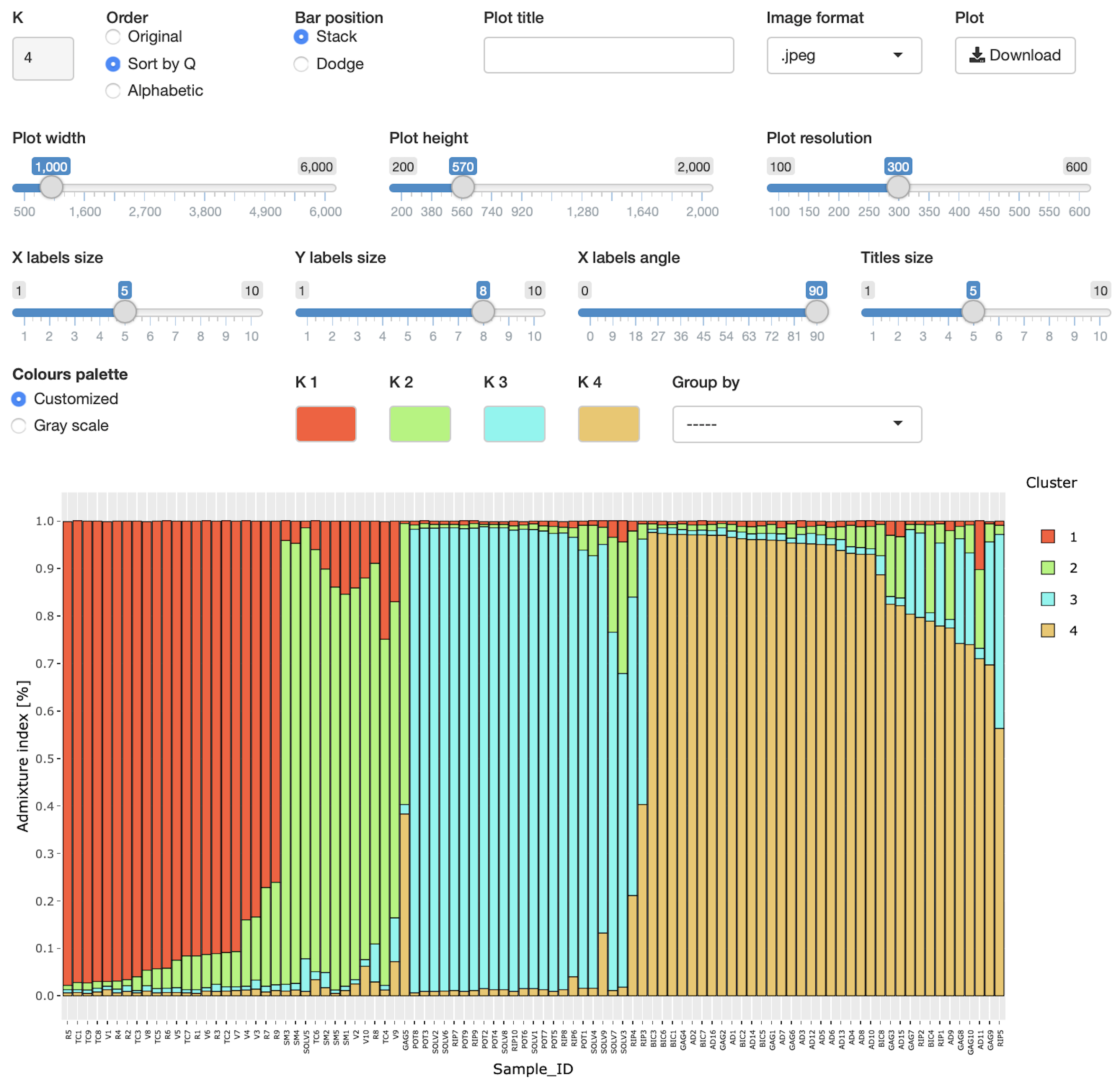

Supplement: S2 Fig — (TIFF) [file pone.0229330.s003.tiff]

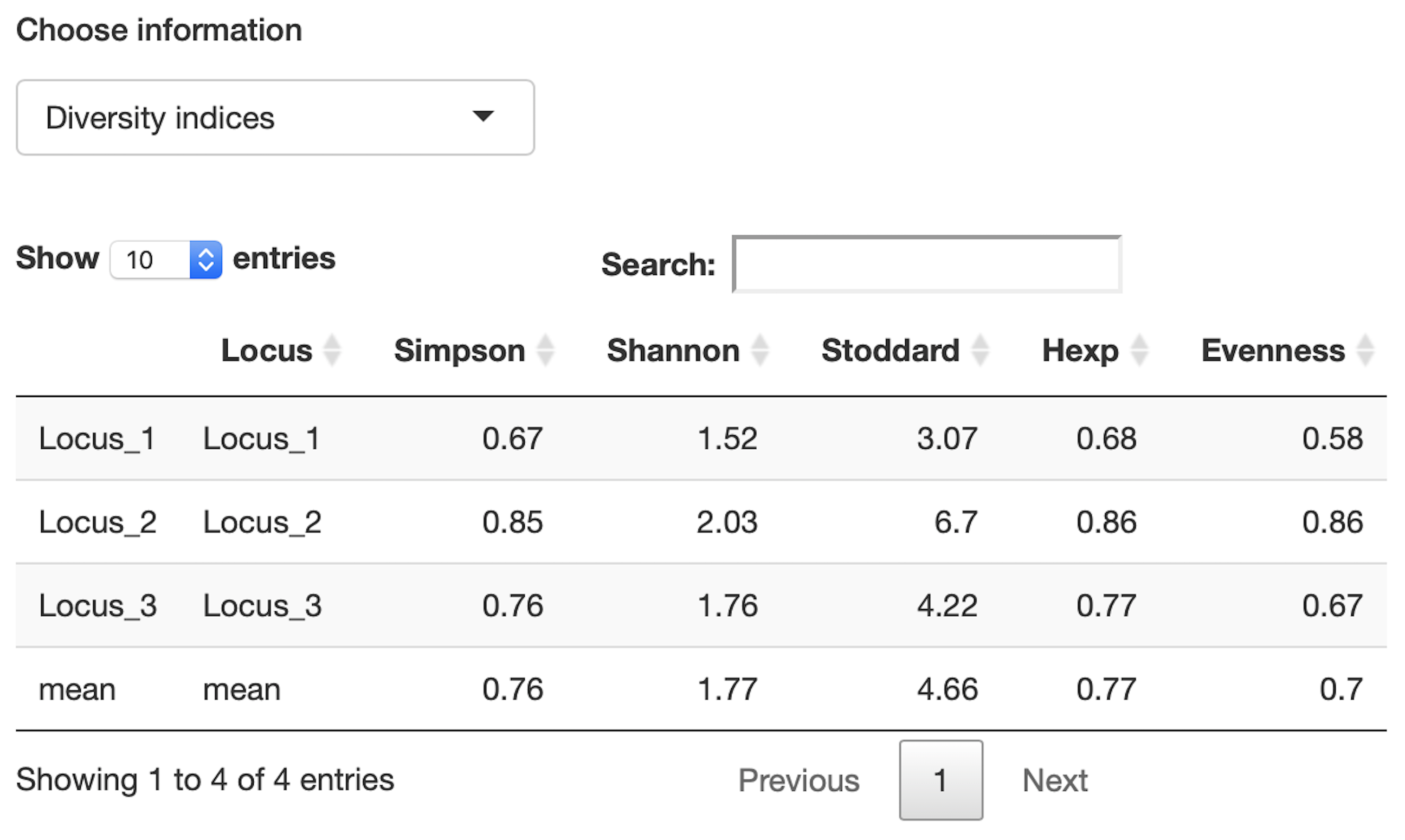

Supplement: S3 Fig — (TIFF) [file pone.0229330.s004.tiff]

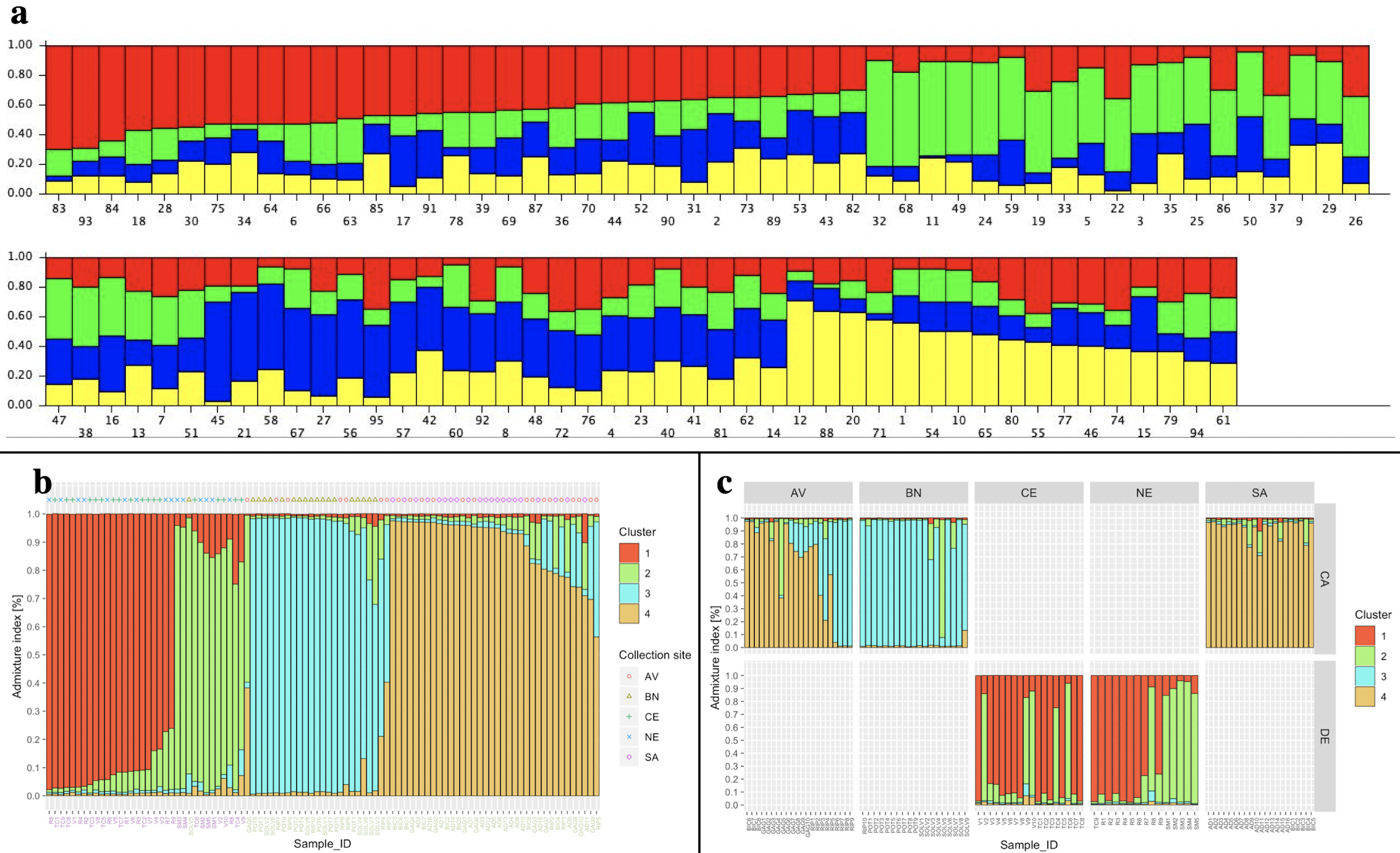

Supplement: S4 Fig — (TIFF) [file pone.0229330.s005.tiff]
